# Effect of vanadium pentoxide concentration in ZnO/V_2_O_5_ nanostructured composite thin films for toluene detection

**DOI:** 10.1039/c9ra02356a

**Published:** 2019-05-28

**Authors:** P. Nagaraju, Y. Vijayakumar, M. V. Ramana Reddy, U. P. Deshpande

**Affiliations:** Nanosensor Research Laboratory, Department of Physics, CMR Technical Campus Hyderabad India 501401 nagarajuphysics@gmail.com; Department of Physics, Osmania University Hyderabad 500007 India; UGC-DAE-CSR Indore Madhyapradesh India 452017

## Abstract

ZnO/V_2_O_5_ nanocomposite thin films were synthesised by the spray pyrolysis technique with optimised deposition parameters by varying the concentration of vanadium pentoxide. The X-ray diffraction results showed that the ZnO/V_2_O_5_ nanocomposite thin films have a Wurtzite-type hexagonal ZnO structure. We attained crystal phases at all concentrations. These results indicated that the two crystal phases of pure zinc oxide and vanadium pentoxide exist together within the composite thin film matrix. The morphology was investigated with field emission scanning electron microscopy and transmission electron microscopy (TEM). The microstructures of the deposited thin films were confirmed by Raman spectroscopy. The optical characterizations of the prepared samples were investigated by using a UV-vis spectrophotometer. X-ray photoelectron spectroscopy (XPS) was carried out to confirm the oxidation states of the elements existing on the surface of the composite thin films. The gas-sensing properties of the composite thin films towards toluene gas were studied at the temperature of 27 °C. The sensing mechanism for toluene gas was reported; the response and recovery times were determined from the transient response curve and were found to be 24 s and 28 s, respectively, for the optimised composite film.

## Abstract 2

ZnO/V_2_O_5_ nanocomposite thin films were synthesised by the spray pyrolysis technique with optimised deposition parameters by varying the concentration of vanadium pentoxide.

## Graphical abstract

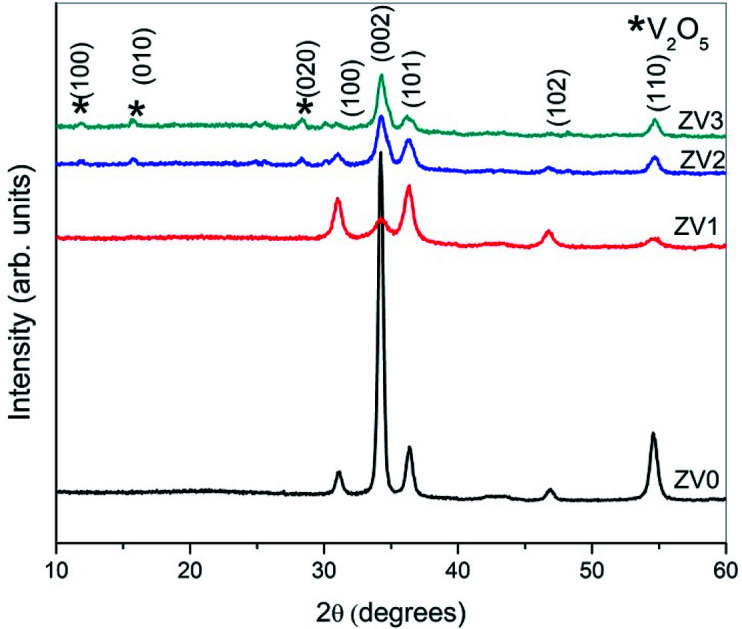

## 1. Introduction

Metal oxide semiconductors are considered as the most important potential candidates due to their versatile applications in solar cells and different transducers such as quartz crystal microbalances, surface acoustic wave (SAW) devices, and photodetectors as well as electronic, optoelectronic, spintronic and chemiresistive gas sensor devices.^1–3^ Among all metal oxides, zinc oxide (ZnO) is a versatile material with a Wurtzite-type structure; it possesses great and distinctive characteristics including a large band gap (3.4 eV), a thermodynamically stable phase and large exciton binding energy, and low toxicity,^4^ due to which it is a flexible material for many industrial and gas sensing applications. Apart from ZnO, vanadium pentoxide (V_2_O_5_) is also a very useful semiconductor oxide material, which is used as an oxidising agent and amphoteric oxide material for industrial applications. It is a major compound of vanadium; it is a primary precursor to the alloys of vanadium and is extensively used as an industrial catalyst.^5,6^ The production of composite materials is an essential technique to control the applications of semiconductors. The preparation of composite materials with two transition metal oxide semiconductors possessing different energy band gap values provides an approach to attain more efficient charge separation, an increased lifetime of the charge carriers and an increased interfacial charge transfer to the adsorbed species, thereby enhancing their gas-sensing properties.^7^ These materials have many advantages in comparison to other materials; the nanowire composite anodes may exhibit a comparable electrochemical performance. A layer-by-layer technique based on the electrostatic attraction between the charged species has been widely used to synthesize polymeric multicomposites, inorganic and hybrid hollow spheres, polymer nanotubes and core–shell nanostructures.^8^ Composite metal oxides manifest various types of morphologies, leading to different properties; thus, they find many further applications such as in lithium-ion batteries, dye-sensitized solar cells, photocatalysis, and gas sensing. Various preparation techniques offer the probability of preparing numerous inherent compounds, which can enable the synthesis and provide a basis for nanostructure-sensitive thin films. Many methods have been employed to deposit composite and pure ZnO thin films, and these include pulsed laser deposition, electron beam evaporation, RF-magnetron sputtering and spray pyrolysis.^9–12^ Among these methods, spray pyrolysis is an easy and low-cost technique. This technique has been extensively studied for the deposition of hard and durable materials for large-area applications. The combinations of structural, optical, electrical and chemical properties have been attained in nanocomposite thin films prepared by the spray pyrolysis technique by varying the deposition parameters. In fact, the stoichiometry and morphology of the nanocomposite thin films can be tailored to suit a distinct application.

Gas sensors are electronic devices that are fabricated to detect the concentration of toxic gases existing in the environment. Gas sensors play an important role in many technological fields, *e.g.*, fuel emission, industry control, household security, and environmental pollution monitoring.^13–15^ Nowadays, gas sensors are very popular and are used in chemical laboratories, hospitals, industries, and almost every technical installation. In the recent past, different types of gas-sensing materials were designed depending on various sensing elements and transduction principles. Among all these materials for gas sensing, chemiresistive composite metal oxide semiconductors are the most promising materials for the detection of low concentrations of hazardous volatile organic compounds (VOCs). Among VOCs, toluene is a colourless organic compound with low vapour pressure and it is the most significant pollutant in the environment. As per the recommendations of the Occupational Safety and Health Administration (OSHA), the permissible exposure limit (PEL) for toluene is 100 ppm of the short time exposure limit. It is found to be associated with nasopharyngeal cancer, asthma, and multiple subjective health issues. Also, toluene can be considered as a very important biomarker for lung cancer.^16^

To the best of our knowledge and belief, this is the first attempt to investigate ZnO/V_2_O_5_ nanocomposite thin films deposited using the spray pyrolysis technique for the detection of toluene at a temperature of 27 °C.

## 2. Experimental techniques

Pure ZnO and ZnO/V_2_O_5_ composite thin films were deposited onto pre-cleaned microscope glass substrates (Blue star, India) using the spray pyrolysis technique (HO-TH-04BT, Holmarc India). For the pure ZnO (ZV0) thin films, zinc acetate dihydrate (Sigma-Aldrich, 99% purity) was dissolved in deionised water to obtain 0.1 M concentration, and few drops of acetic acid were added to obtain a clear and stable solution. For the deposition of the films, compressed and filtered air was used to atomise the solution with a flow rate of 1 mL min^−1^ for 15 min. The substrate temperature was fixed at 425 °C and was maintained within ± 3 °C by using a temperature controller, and the nozzle-substrate distance (NSD) was maintained at 25 cm. For the composite films, a V_2_O_5_ precursor solution was prepared with ammonium vanadate (Sigma Aldrich, 99%) in deionised water. The solution of the composites was prepared by adding the precursors ZnO and V_2_O_5_ at molar ratios of 90 : 10 (ZV1), 80 : 20 (ZV2), and 70 : 30 (ZV3), and the other deposition conditions were the same as those of the pure films.

### 2.1. Characterization techniques

The thicknesses of all thin films were measured using a weight balance method, which has been discussed elsewhere.^17^ The structural properties of the pure and composite thin films were analysed by using a grazing incidence X-ray diffraction system (Brucker D8-Discover X-ray diffractometer) with Cu Kα radiation (*λ* = 0.15418 nm) in the range of 20–80° with 1° min^−1^ as the scanning speed. Raman spectra were recorded in the Raman shift range of 100–1000 cm^−1^ with backscattering geometry using a 9 mW semiconductor diode laser (473 nm) as an excitation source. Transmission electron microscopy (TEM) was performed using TEM model FEI TECNAI G2 S-Twin with an operating voltage of 200 kV. The TEM studies were executed in both the diffraction and image modes. The surface morphology of the composite thin film samples was investigated by field emission scanning electron microscopy (FESEM) and the elemental analysis of the film was carried out with energy dispersive X-ray analysis (EDAX) using FEI NoVa NanoSEM 450 operated at 18 kV. Compositional analysis was performed using a Bruker-made X-Flash 6130 EDS attachment and Esprit software. The optical transmittance was determined using an ultraviolet-visible-near-infrared (UV-vis-NIR) double-beam spectrophotometer (PerkinElmer, USA, Lambda 950) by scanning wavelengths in the range of 300 nm to 1000 nm with a step size of 2 nm. X-ray photoelectron spectroscopy studies were performed on an Omicron energy analyzer (EA-125) with Al Kα radiation, and photon energy of 1.486 keV was used as the X-ray source at operating conditions of 15 kV and 10 mA.

### 2.2. Gas sensing measurements

We prepared 1 cm × 1 cm thin films on a glass substrate for gas sensing measurements. Electrical contacts were made by pasting high pure silver paste and drying in a hot air oven at 150 °C for one hour. The measurements were obtained in the static mode using a custom-made air-sealed stainless-steel gas-testing chamber of 5 L volume containing a thermocouple, heater and probes along with an electrometer (Keithley 6517B, USA). The desired concentration of toluene solution was injected into the test chamber using a micro syringe. The response (*S*) of the test gas was calculated using the relation *S* = *R*_a_/*R*_g_, where *R*_a_ is the resistance in the dry air atmosphere and *R*_g_ is the resistance in the presence of the test gas.^18^ We maintained the relative humidity in the chamber at 60% using a digital humidity controller (Humitherm, India) during the gas sensing measurements.

## 3. Results and discussion

### 3.1. X-ray diffraction (XRD)

**Fig. 1:**
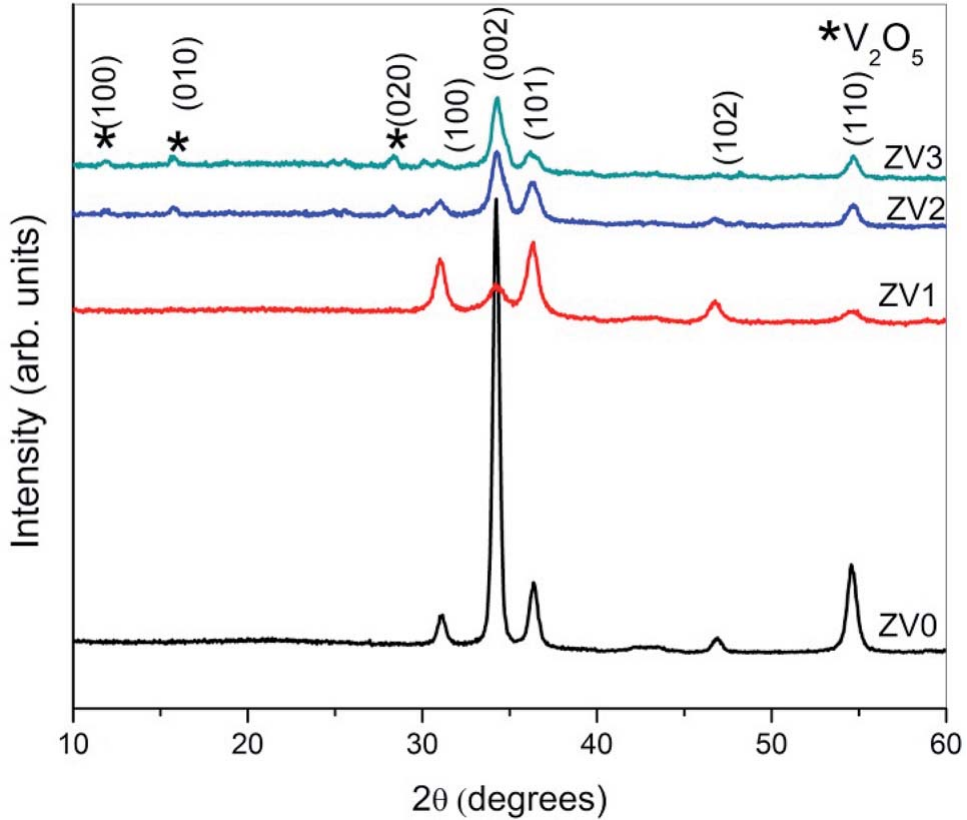
X-ray diffraction patterns of pure ZnO and ZnO/V_2_O_5_ composites.

The crystal structure, orientation and phase purity of pure ZnO and ZnO/V_2_O_5_ composite thin films were analysed by using X-ray diffraction, as shown in Fig. 1. The results show that the crystallinity of the films varies depending on the molar concentration of vanadium pentoxide. From the XRD spectrum, it is clear that all the films are polycrystalline in nature. The polycrystalline zinc oxide peaks in the patterns were identified as (100), (002), (101), (102) and (110), whereas the vanadium pentoxide peaks were identified as (100), (010) and (020). These peaks are in accordance with the values of JCPDS file number 089-0510 for zinc oxide and 00-053-0538 for vanadium pentoxide. It is known that if vanadium pentoxide is incorporated into the ZnO crystal lattice, the XRD peaks may shift by a small angle due to the radius mismatch of the vanadium ion (0.059 nm) and zinc ion (0.074 nm). We observed that the XRD peaks did not shift, which indicated the formation of ZnO/V_2_O_5_ composites. In the composite thin films, zinc oxide and vanadium pentoxide exhibited hexagonal wurtzite and orthorhombic structures, respectively. The intensity of the (002) plane of the ZV0 sample was maximum; also, for the ZV1 sample, its intensity decreased and after that, it slightly increased for ZV2 and ZV3 samples, indicating the growth along this phase. The introduction of vanadium pentoxide at a given substrate temperature may obstruct the crystallization and crystal growth of zinc oxide. The lattice constants (*a* and *c*) of different crystal planes (*hkl*) were determined using the following equation.^19^ The lattice constants of all the samples are tabulated in Table 1.1
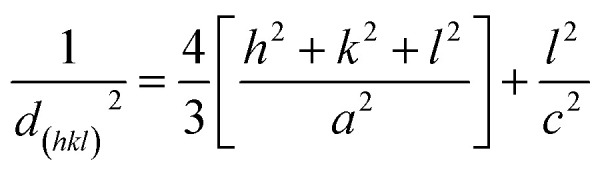


**Table 1:** Structural parameters of pure ZnO and ZnO/V_2_O_5_ composites

S. no	Sample	Lattice constant ‘*c*’ (nm)	Lattice constant ‘*a*’ (nm)	Crystallite size (*D*) (nm)	Dislocation density (*δ*) (×10^−3^ nm^3^)	Strain (×10^−3^)
1	ZV0	0.524	0.332	22.056	2.055	1.640
2	ZV1	0.525	0.331	22.051	2.056	1.641
3	ZV2	0.524	0.332	24.510	1.664	1.476
4	ZV3	0.522	0.332	31.520	1.006	1.148

The crystallite sizes of all the samples were determined with the Scherrer's formula^20^ using the almost intense peak (002), where the crystal sizes were between 31.52 nm and 22.05 nm as the molar concentration of V_2_O_5_ increased.2
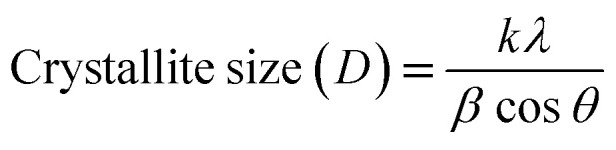
Here, *λ* is the X-ray wavelength (0.15418 nm), *k* is a constant, which is equal to 0.94, *β* is the peak full-width at half maximum, and *θ* is the peak position. The calculated crystallite sizes of all samples are tabulated in Table 1.

The strain and dislocation densities of the pure and composite thin films were determined using the following relations:3
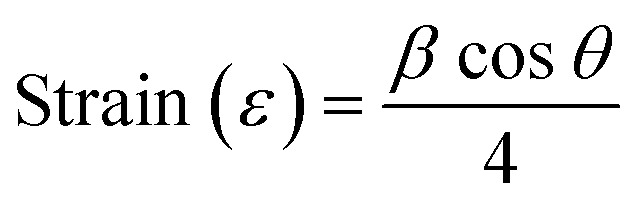
4
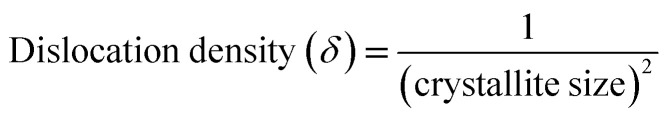


### 3.2. Raman spectroscopic analysis

**Fig. 2:**
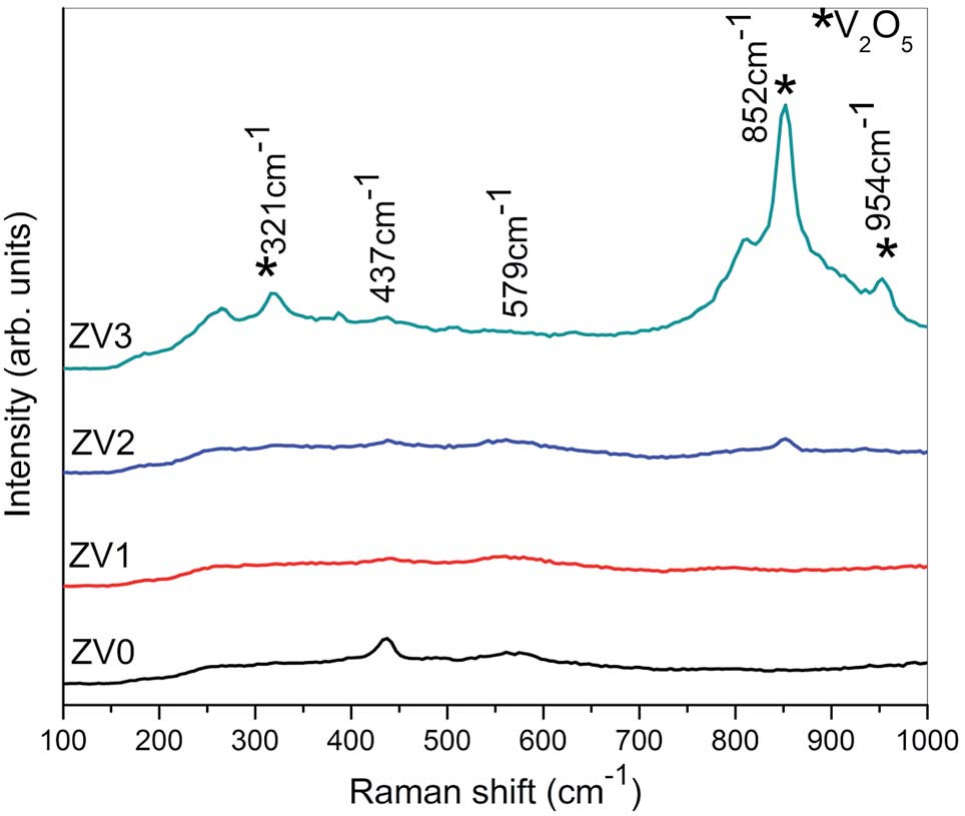
Raman spectra of pure ZnO and ZnO/V_2_O_5_ composites.

Raman spectroscopy is a non-destructive sophisticated technique for analysing composite materials and for extracting useful information on the properties of nanostructures. For the V0 sample, the Raman spectrum shows a vibrational mode at 378 cm^−1^ related to E1 (TO) and a vibrational mode at 437 cm^−1^ related to *E*_2_ as well as transverse oscillation (TO) at 988 cm^−1^;^21^ these correspond to the bands characteristic to the hexagonal wurtzite structure belonging to the space group *C*_6*v*_^4^ with two formula units per primitive cell of zinc oxide. The vibrational mode at 579 cm^−1^ is due to the longitudinal optical (LO) phonon mode of zinc oxide having E1 symmetry. For the V1, V2, V3 samples, the mode at 265 cm^−1^ corresponds to the V–O–V bending mode and also, the other modes at higher wavenumbers of 852 cm^−1^ and 954 cm^−1^ confirm the composite formation of ZnO/V_2_O_5_ thin films (Fig. 2).

### 3.3. Transmission electron microscopy studies

**Fig. 3:**
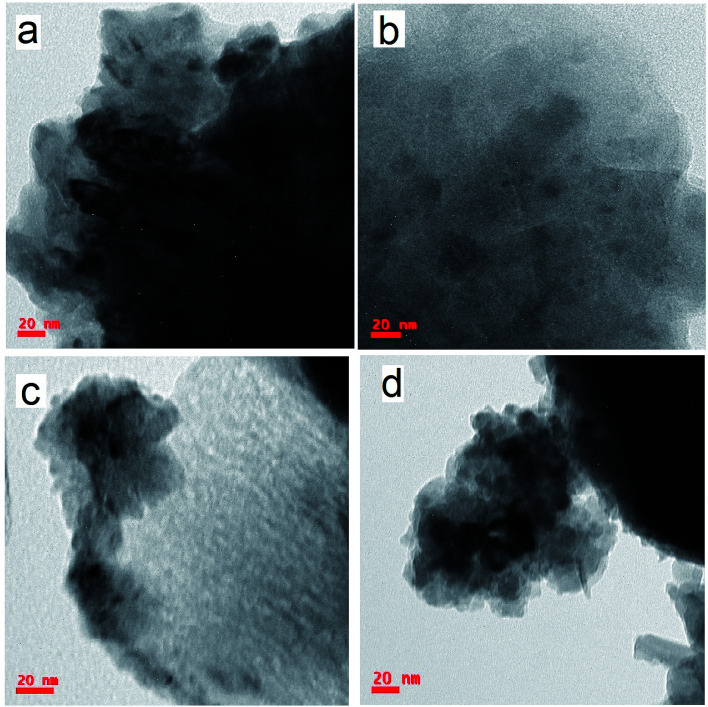
Transmission electron microscopy images of (a) ZV0, (b) ZV1, (c) ZV2, and (d) ZV3.

**Fig. 4:**
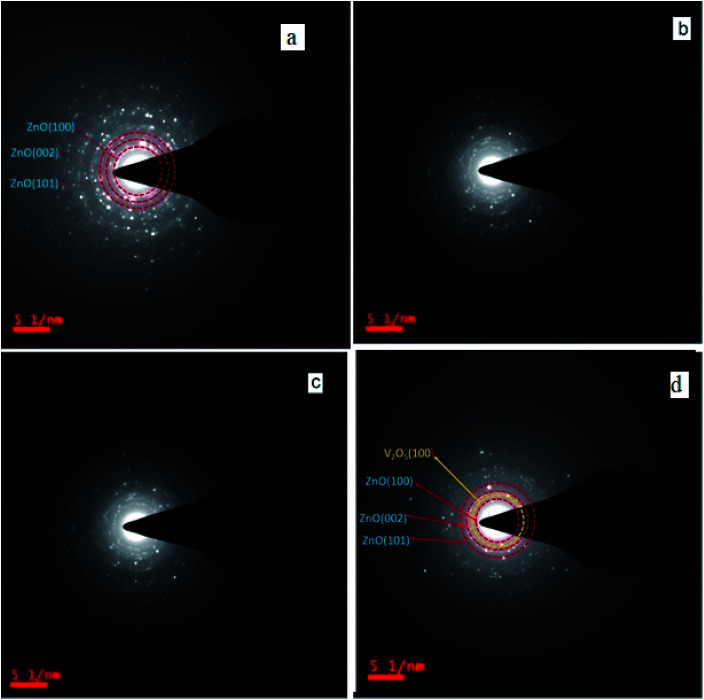
SAED images of (a) ZV0, (b) ZV1, (c) ZV2, and (d) ZV3.

Transmission electron microscopy (TEM) is used to investigate the microstructural properties of composite thin films such as crystallite size, crystal structure, morphology, defects in the material, crystal phases and composition. It enables the determination of the interplanar spacing, *d*, from the rings of the selected area electron diffraction (SAED) pattern. Fig. 3 depicts the transmission electron microscopy (TEM) images of the pure and composite thin films, which clearly indicate the presence of two different materials in the composite matrix. Fig. 4 shows the selected area electron diffraction (SAED) patterns of the nanostructured ZnO/V_2_O_5_ composite thin films. The diffraction patterns consist of concentric rings, which imply polycrystalline nature with distinct (100), (002), and (101) planes of the pure ZV0 sample and also the (100) plane, corresponding to vanadium oxide, for all other composite samples; these results are in agreement with the X-ray diffraction observations. The interplanar spacing, *d*, can be determined using the following equation,^22^ and the corresponding values are tabulated in Table 2:5
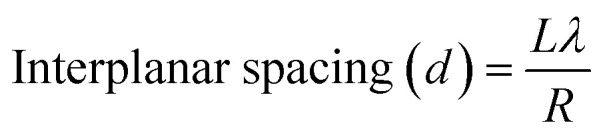
Here, *L* is the length of the camera, which is equal to100 mm, *λ* is the electron wavelength, and *R* is the radius of the ring; it is measured from the central bright. For an operating voltage of 200 kV, the corresponding electron wavelength is 0.0027 nm. The determined values of interplanar spacing (*d*) from the above eqn (5) match well with those of the X-ray diffraction studies.

**Table 2:** Interplanar spacings (*d*) of pure ZnO and ZnO/V_2_O_5_ composites

S. no	Sample	Interplanar spacing *d*_100_ (nm)	
		From XRD studies	From TEM studies
1	ZV0	0.28	0.31
2	ZV1	0.27	0.30
3	ZV2	0.28	0.27
4	ZV3	0.28	0.28

### 3.4. Optical properties

**Fig. 5:**
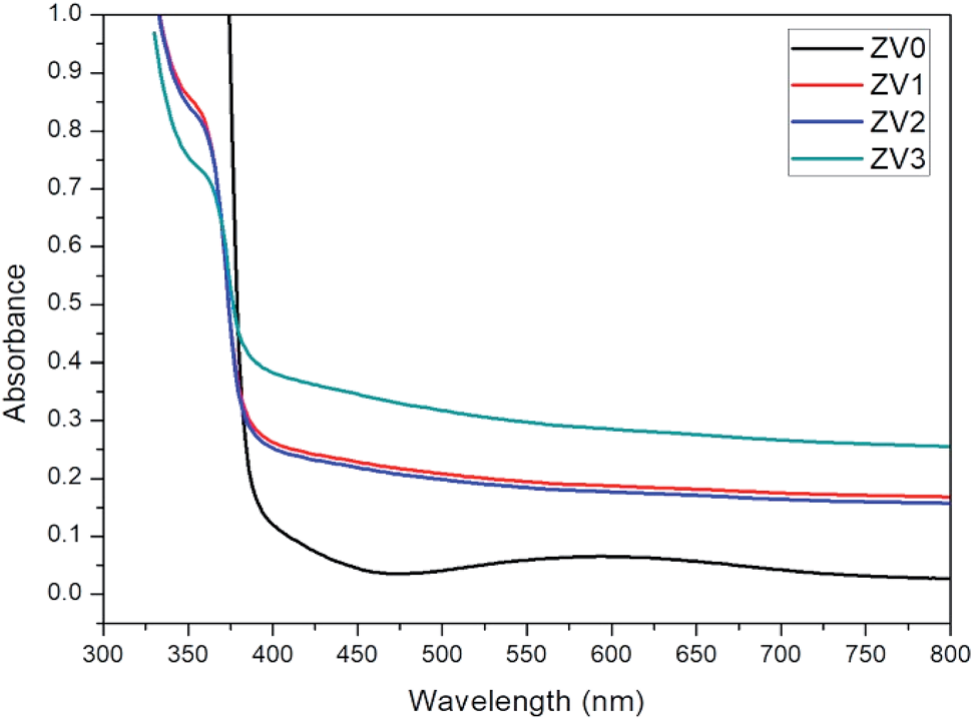
Absorbance spectra of pure ZnO and ZnO/V_2_O_5_ composite thin films.

Fig. 5 shows the optical absorption spectra of pure ZnO and ZnO/V_2_O_5_ composite thin films. An absorption edge at about 383 nm is only observed for the ZV0 sample, which is characteristic of zinc oxide. An absorption edge slightly increases from 360 nm to 367 nm as the V_2_O_5_ concentration increases. This result is consistent with the XRD observations. The band gaps of nanostructured pure ZnO and ZnO/V_2_O_5_ composite thin films were determined using the following Tauc formula:^23^

6 *αhν* = *B*(*hv* − *E*_g_)

Here, *α* = absorption coefficient, *ν* = frequency of the photon, *h* = Planck constant, *E*_g_ = optical band gap.

**Fig. 6:**
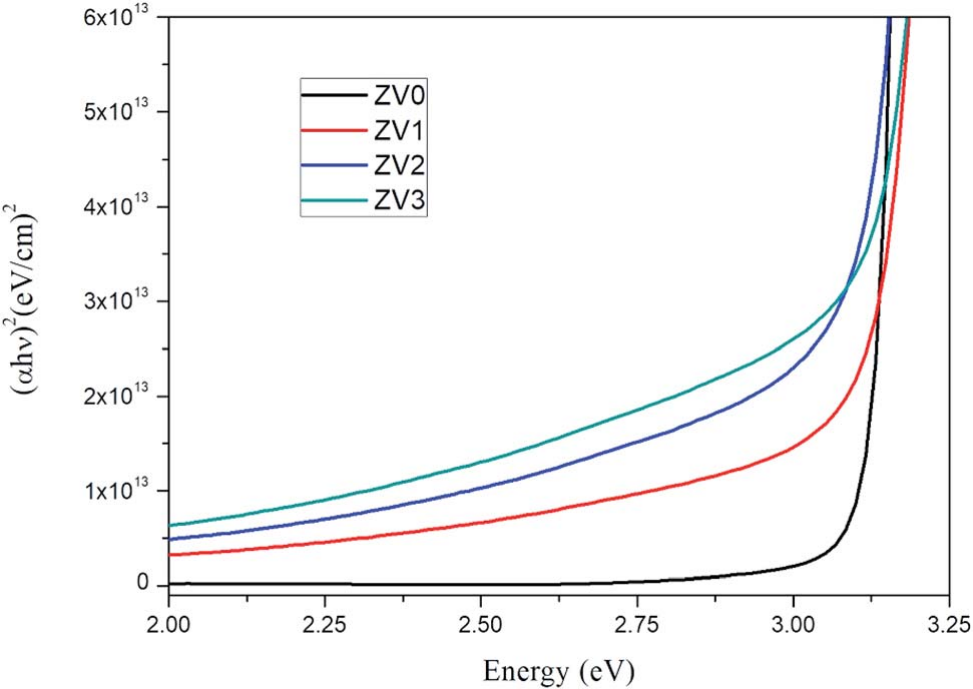
Tauc plots of pure ZnO and ZnO/V_2_O_5_ composite thin films.

The plots of (*αhν*)^2^*versus* the energy of the photon (*hν*) of pure ZnO and ZnO/V_2_O_5_ composite thin films are depicted in Fig. 6. The band gap (*E*_g_) was determined by extrapolating the linear portions to the horizontal photon energy axis.^24,25^ The calculated optical band gaps of the pure ZnO and ZnO/V_2_O_5_ composite thin films are tabulated in Table 3. The band gap decreased as the concentration of vanadium pentoxide increased. The decrease in the bandgap suggests that a small amount of vanadium ion impurities is incorporated in the V_2_O_5_ and (or) ZnO layers and the thickness of the film decreased on increasing the concentrations of vanadium pentoxide.

**Table 3:** Optical bandgaps of pure ZnO and ZnO/V_2_O_5_ composite thin films

S. no	Sample	Thickness of the film (nm)	Optical band gap (eV)
1	ZV0	275	3.12
2	ZV1	250	3.09
3	ZV2	228	3.03
4	ZV3	212	2.97

### 3.5. Field emission scanning electron microscopy (FESEM) and energy-dispersive X-ray spectroscopy (EDX) analyses

**Fig. 7:**
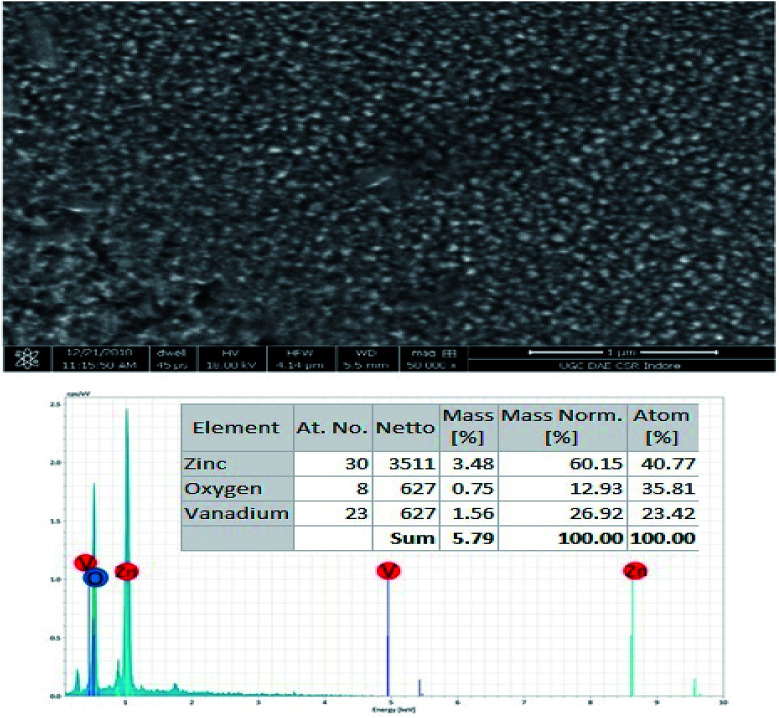
FESEM and EDX analyses of the ZnO/V_2_O_5_ composite thin films.

The FESEM analysis, grain formation and EDX spectra of the ZV3 composite thin films are depicted in Fig. 7. It is clear from the figure that the sizes of the grains are found to be less than 30 nm; the surface morphologies are more compact and continuous with the presence of closely packed particles, which might be due to the attraction between the V_2_O_5_ and ZnO particles. Hence, it may not affect the mobility of electrons travelling between the grains.^26^ Large agglomerated grains were homogeneously distributed with good adhesion to the substrates. The EDX spectra elucidate that the synthesized composite thin film exhibits the presence of Zn, V, and O only. No other remarkable impurities were observed.

### 3.6. X-ray photoelectron spectroscopy (XPS)

**Fig. 8:**
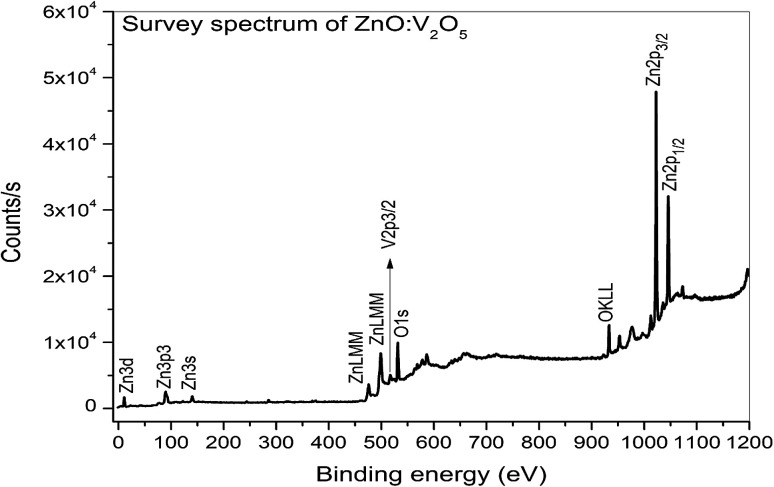
Full XPS survey spectra of ZnO/V_2_O_5_ composite thin films.

**Fig. 9:**
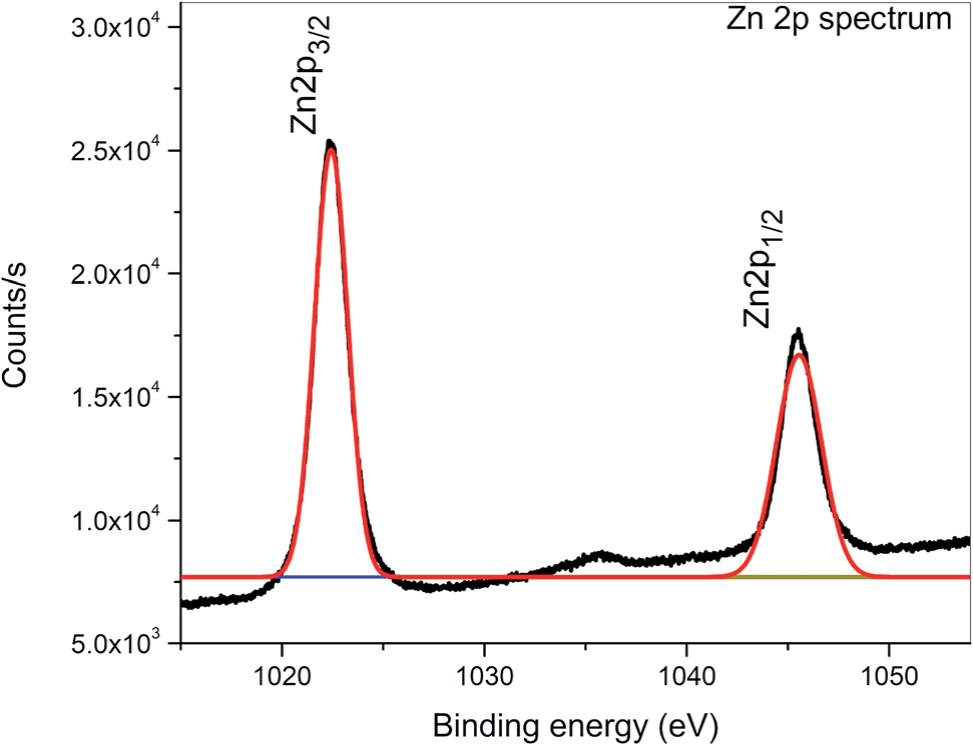
X-ray photoelectron spectrum of Zn 2p.

**Fig. 10:**
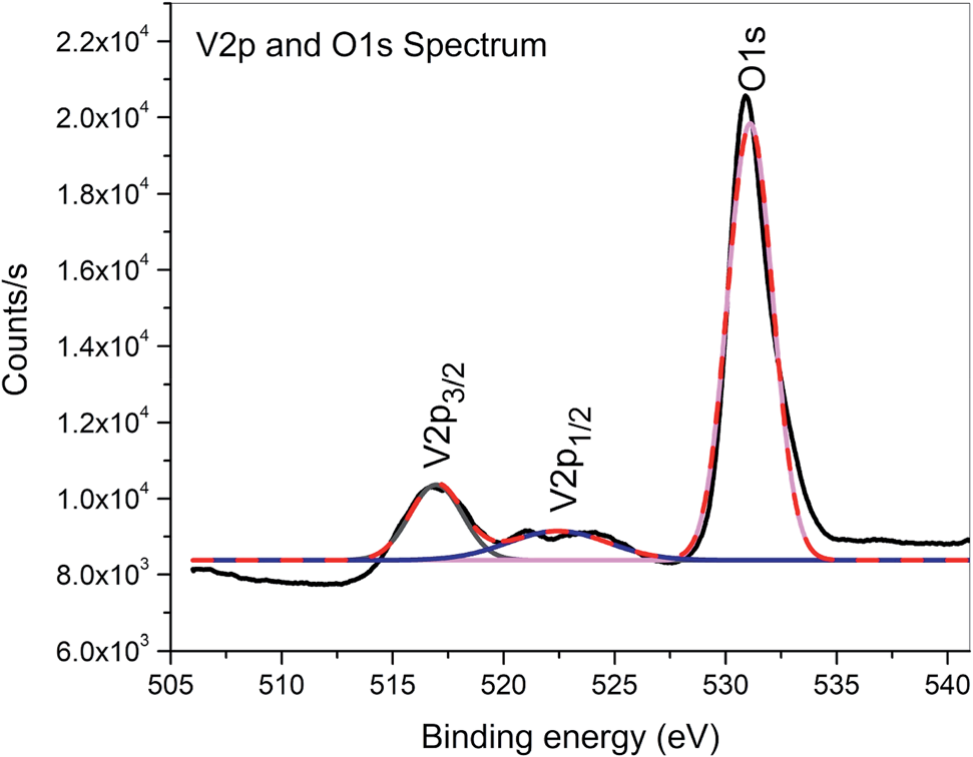
X-ray photoelectron spectra of V 2p and O 1s.

XPS is a surface-sensitive characterization technique, which is adopted to measure the electronic state, elemental composition and chemical bonding of a composite thin film. The distinctive binding energy (eV) peaks matching with all the elements present in the sample, *i.e.*, Zn, V and O were observed in the survey spectrum. The full scan survey spectrum of the ZnO/V_2_O_5_ (ZV3) composite is shown in Fig. 8. In the current investigation, the ZnO/V_2_O_5_ composite thin film has V 2p_3/2_ peak position at 516.65 eV with a Δ*E* value of 14.35 eV, exhibiting a shift to a lower binding energy. The lower binding energy of V 2p_3/2_ and the increase in the Δ*E* value specify the chemical state variation of the V_2_O_5_ compound.^27^ It is therefore considered that the shift originates from the interfacial diffusion of vanadium atoms into the ZnO film since it is known that ZnO is capable of accommodating interstitial vanadium ions.^28,29^Fig. 9 depicts the X-ray photoelectron spectroscopy results of the Zn 2p state. The peak existing at the binding energy of 1021.19 eV confirms that Zn is in the form of Zn^2+^.^30^Fig. 10 shows the fitted X-ray photoelectron spectroscopy results for V 2p and O 1s. The peak position of O 1s is at 530.10 eV, indicating the main chemical oxidation states of the lattice oxygen in the ZnO/V_2_O_5_ composite thin film.^31^

### 3.7. Gas sensing characteristics

Due to the catalytic behaviour of ZnO for toluene, the ZnO/V_2_O_5_ composites could enhance the sensitivity of the sensor towards toluene. The sensing mechanism of chemi-resistive gas sensors mainly depends on the change in electrical resistivity contributed by interactions between the surface of the sensor and test gases. In detail, when the ZnO/V_2_O_5_ composite sensing element was exposed to air, adsorbed oxygen molecules captured electrons from the surface of the sensor; hence, chemi-adsorbed oxygen species were generated. These chemi-adsorbed oxygen species resulted in the formation of a depletion layer at the grain boundaries. Also, when the composite sensors were exposed to a toluene gas atmosphere, the toluene molecules were adsorbed onto the composite thin film surface. The reductive toluene vapours reacted with the chemically adsorbed oxygen ions, promoting the captured electron transition in the sensing mechanism.^32^ This process can be explained as follows:C_6_H_5_CH_3_ + O^2−^ → C_6_H_5_CHO^−^ + H_2_O + e^−^C_6_H_5_CHO^−^ → C_6_H_5_CHO + e^−^

**Fig. 11:**
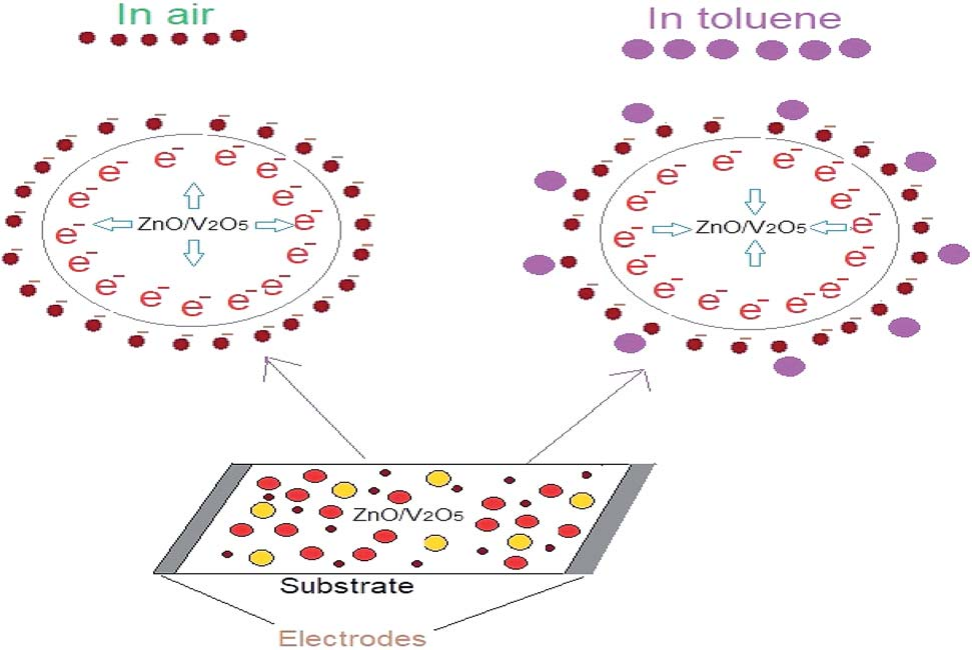
Schematic illustration of the toluene sensing mechanism of the ZnO/V_2_O_5_-based sensors.

The reduction and oxidation reactions led to decrease in the electrical potential across the depletion layer and then, decrease in the resistance of the composite sensor was observed. A typical toluene sensing mechanism is shown in Fig. 11.

**Fig. 12:**
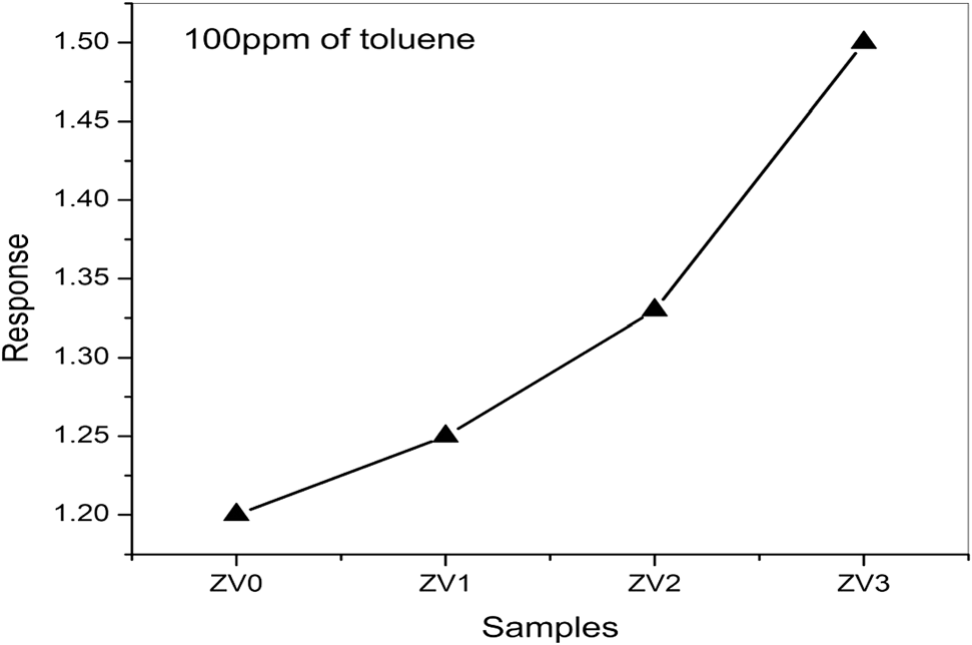
Gas sensing performances of pure ZnO and ZnO/V_2_O_5_ composite thin films.

**Fig. 13:**
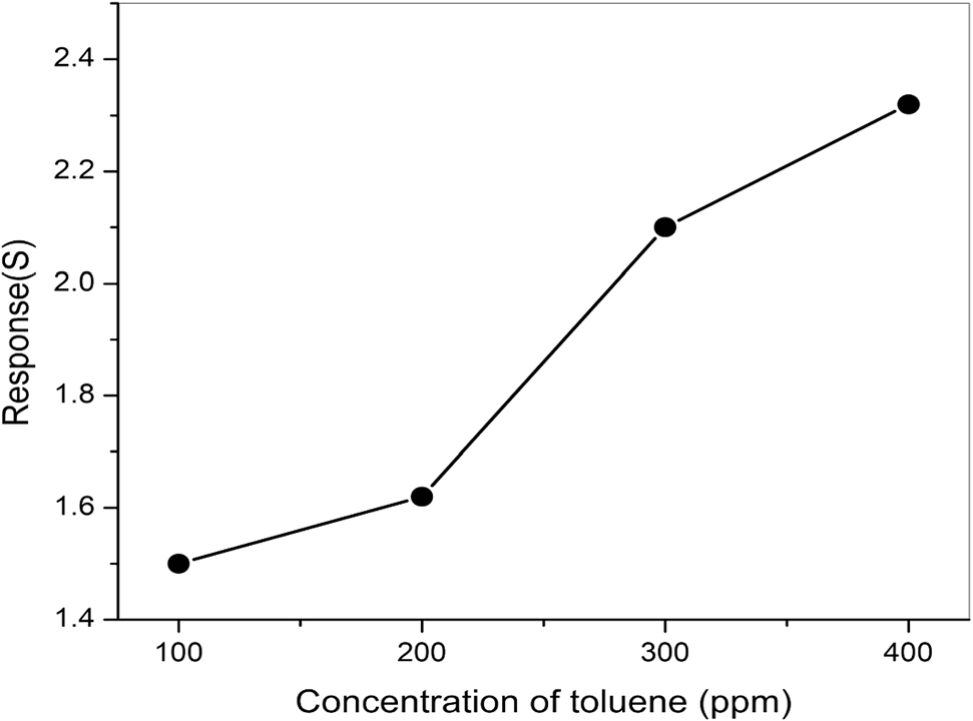
Responses of ZnO/V_2_O_5_ composite film to different concentrations of toluene at 27 °C.

The responses of pure ZnO and different molar concentrations of ZnO/V_2_O_5_ composite thin films towards toluene were measured at a temperature of 27 °C. From Fig. 12, it is clear that the response towards toluene is notably improved by the addition of V_2_O_5_ to the ZnO thin film. The composite thin-film sensor ZV3 showed the highest response, which can be attributed to the increase in the molar concentration of vanadium pentoxide in the composite matrix. This led to enhancement in the chemically adsorbed oxygen, consequently leading to an increase in the electron carrier concentration, which is beneficial to enhance the gas sensing properties of the composite sensor.^33^ The results for the ZV3 sample showed that the vanadium pentoxide content should be optimised to acquire the best toluene-sensing properties in the ZnO/V_2_O_5_ thin film. Fig. 13 depicts the dynamic responses towards the ZV3 sample with different concentrations of toluene at 27 °C. The responses of the ZV3 sample increased obviously with the increase in toluene concentration in the range of 100–400 ppm and exhibited good linearity with the increase in toluene concentration. This linear correlation of the sensitivity and concentration suggests that the ZV3 sample can sense toluene gas for many applications.^34^

**Fig. 14:**
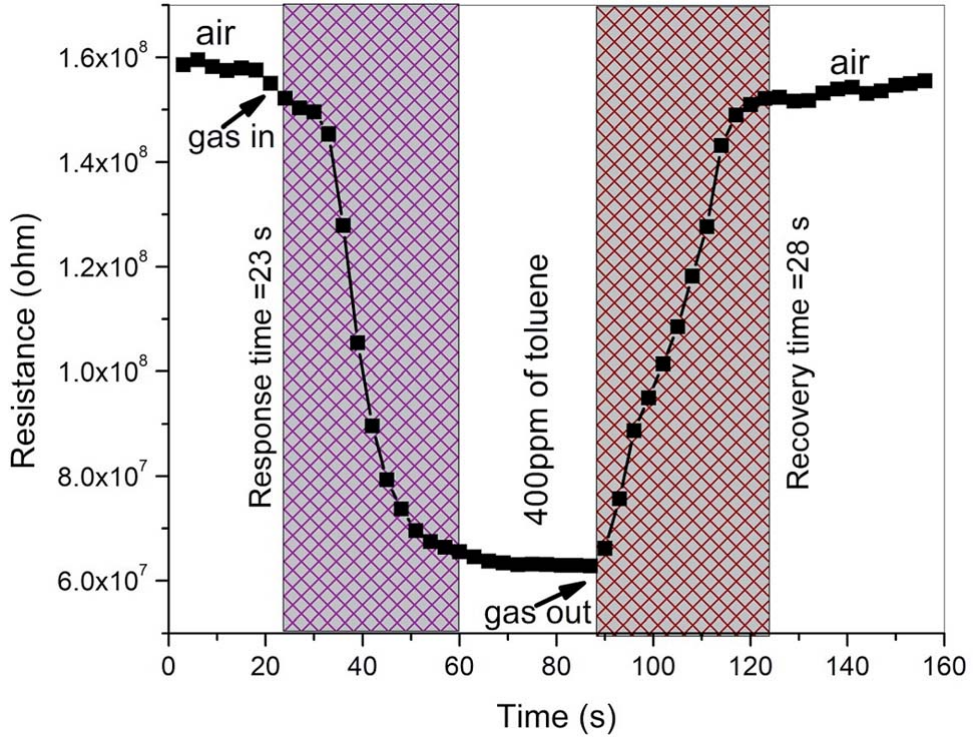
Transient response curve of the ZnO/V_2_O_5_ composite thin films.

Fig. 14 shows the transient response of the ZnO/V_2_O_5_ composite sensor at a concentration of 400 ppm of toluene at the temperature of 27 °C. The response time is described as the time needed for attaining 90% of the baseline resistance after the test gas exposure, and the recovery time is described as the time required for the sensor to return its resistance to 10% of the baseline resistance after the test gas is removed. The ZnO/V_2_O_5_ composite is an n-type semiconductor; hence, in the presence of a reducing gas, the sensor resistance decreased to an equilibrium value and returned to the baseline value after ventilating the test gas.^35^ From the transient response curve, it is found that the response and recovery times are 23 s and 28 s, respectively. For majority of the sensors, the response time is quicker in comparison with the recovery time. This may be due to the crystallite size and surface morphology of the composite film sensor. In the present investigation, the smaller response time might be due to the faster reaction rate between the crystallites on the surface of the thin composite films, which can result in the adsorption of a large number of oxygen molecules, consecutively producing a faster response at the point of the gas inlet. The response and recovery times of different composite materials reported in the literature towards toluene are tabulated in Table 4. It is clear that our composite sensor shows a good response at 27 °C.

**Table 4:** Response/recovery times of different composite materials towards toluene

S. no	Materials	Temperature (°C)	Response time (s)	Recovery time (s)	Ref.
1	Au/ZnO nanoparticles	377	NA	300	36
2	Co_3_O_4_ nanorods	200	90	55	37
3	Fe_2_O_3_/SnO_2_ nanowires	90	20	15	38
4	ZnFe_2_O_4_ nanospheres	300	20	30	39
5	ZnO/V_2_O_5_ thin films	27	23	28	Present work

## 4. Conclusions

ZnO/V_2_O_5_ composite thin films were deposited using the spray pyrolysis technique by varying the concentration of V_2_O_5_ with optimised deposition parameters. All the films were polycrystalline in nature, and the crystallite size increased with the increase in the V_2_O_5_ concentration. Raman spectra also suggested the formation of ZnO/V_2_O_5_ composites with a Wurtzite phase. The chemical states and the electronic structure of the composite thin films were examined by X-ray photoelectron spectroscopy, which indicated the presence of vanadium ions in the ZnO composite matrix. The toluene-sensing properties of the ZnO/V_2_O_5_ composite thin-film sensors were examined, and the effect of V_2_O_5_ concentration in ZnO was discussed. The ZV3 sensor showed the best response towards toluene at 27 °C than the other composite thin-film sensors. The response and recovery times of the ZV3 sensor were determined and reported. The present results show that the ZnO/V_2_O_5_ composite thin films are potential materials to detect toluene gas.

## Conflicts of interest

There are no conflicts to declare.

## Supplementary Material
